# Post-Acute Rehabilitation in Persons With Dementia: Does It Make a Difference?

**DOI:** 10.1093/geroni/igaa057.611

**Published:** 2020-12-16

**Authors:** Jane Flanagan, Marie Boltz, Ming Ji

**Affiliations:** 1 Boston College, Chestnut Hill, Massachusetts, United States; 2 Penn State University, University Park, Pennsylvania, United States; 3 U. South Florida, Tampa, Florida, United States

## Abstract

Persons with dementia are about two times more likely to be hospitalized than their peers who are cognitively healthy. These individuals are frequently discharged to skilled nursing facilities or nursing home settings, to receive short-term, post-acute, rehabilitative care. The rehabilitative care, Physical Therapy (PT) and Occupational Therapy (OT), provided in skilled nursing facilities (SNFs) aims to restore the person to their pre-hospitalization functional status and assist the person to return home. This study used MDS assessment data of 6396 people, age 65 years and older with dementia, admitted to SNFs in 2013 from acute care hospitals in Massachusetts to assess the effects of OT and PT on the change in physical function of nursing home residents admitted to the nursing home after hospitalization. Multiple linear regression analyses. The sample was mostly female (64.1%), non-Hispanic (98.86%), and white (93.71%), with a mean age of 85.3 (SD=6.85). After controlling for age, gender, race and comorbidities, and delirium, rehabilitation interventions (OT, PT or OT+PT) did not have any significant effect on changes in physical function among residents with dementia (p for OT = 0.14; p for PT=0.59; p for OT+PT:= 0.32). Additionally, non-white residents had poorer function at three months (β =1.86, 95% CI:-3.57- -0.16). The results indicate for persons with dementia admitted to SNFs, OT, PT or OT+PT did not lead to a significant improvement physical function. More innovative and effective interventions should be developed to improve physical function in persons with dementia post-hospitalization.

